# Notes and News

**Published:** 1888-09-08

**Authors:** 


### Notes and News.

**Collected and Communicated.**

A man named Wiedhoft died in St. Bartholomew's Hospital on Monday, from injuries caused by a fall from a scaffold erected at the "Sun" offices, in Threadneedle street.

In aid of the funds of the Royal Hospital for Diseases of the Chest, City Road, an evening concert is to be given by the Alpine Club, at the Myddelton Hall, Islington, on Monday, September 10th.

The Rev. S. A. Barnett, M.A., vicar of St. Jude's, Whitechapel, and Mrs. Barnett, have in the press a joint volume of essays on social reform, under the title of "Practicable Socialism."

The late Miss Annie Lowther, Moresby House, near Whitehaven, a relative of Lord Lonsdale, has left legacies of £1,000 to the Whitehaven and West Cumberland Infirmary, and £1,000 to the Royal National Lifeboat Institution. Various local charities also receive smaller legacies.

The patients at the Convalescent Home, Saltburn-by-the-Sea, last week presented the matron, Miss Cox, with a handsome silver hot-water jug, on the occasion of the twenty first anniversary of her appointment to the office. A suitable inscription, setting forth the esteem in which Miss Cox has long been held, was engraved on the memorial gift.

It is expected that the workshop collection of the Hospital Saturday Fund will amount to at least £8,000, making, with the street collection, a total of £13,000, the largest sum ever yet collected in connexion with the Fund. Even this amount falls far short of what the working population of London should feel bound to contribute towards the support of the metropolitan hospitals.

A proposal by the Coopers' Company to remove their old-established and popular grammar school in Ratcliff Highway to East Ham has caused a good deal of regret at the East-end, and the vestrymen of Bromley and Poplar are about to formally petition against the change. It is admitted that the decision rests entirely with the company, but the history of the school, and the benefit it has been to the neighbourhood, are urged as reasons for its retention.

Baths and washhouses are especial desiderata in all large towns. On Saturday a new range of buildings for lavatory purposes was opened in Flask Walk, Hampstead. The baths have been erected by the trustees of the local Wells and Camden Charity at a cost of nearly £3,500. This makes the second set of baths and washhouses provided for the parish by that charity. The Commissioners of Baths and Washhouses appointed by the Hampstead Vestry have erected, and recently opened, a handsome structure, including two large swimming and other baths, in the Finchley New Road.

In London last week 2,401 births and 1,438 deaths were registered. Allowing for increase of population, the births were 300, and the deaths 115, below the average numbers in the corresponding weeks of the last ten years. The annual death-rate per 1,000 from all causes, which had been 15·9, 18·0, and 16·2 in the three preceding weeks, rose again last week to 17·5. During the first eight weeks of the current quarter the death-rate averaged 16·2 per 1,000, and was 4·7 below the mean rate in the corresponding periods of the ten years 1878-87. The 1,438 deaths included 47 from measles, 21 from scarlet fever, 17 from diphtheria, 28 from whooping-cough, 7 from enteric fever, 192 from diarrhœa and dysentery, 7 from cholera and choleraic diarrhœa, and not one from small-pox, typhus, or ill-defined forms of continued fever; thus, 319 deaths were referred to these diseases, being 36 below the corrected average weekly number.

Our readers will learn with regret of the death of Miss Rogers, sister of the Rev. Wm. Rogers, M.A., rector of St. Botolph, Bishopsgate, which took place on the morning of last Thursday, at the Old House, Mickleham, Surrey. The deceased lady was at one time well known in the City, and, until confined to her room two or three years ago by a lingering illness was, with her sister Miss Georgiana Rogers, a regular visitor at the Bishopsgate Schools, and assisted her brother at the social gatherings by means of which the rector of St. Botolph has done so much to promote good feeling in his parish. The funeral took place yesterday, when the remains were laid in the family vault in Mickleham churchyard, in the presence of many relations and friends, the Rev. William Rogers and the Rev. R. H. Hadden conducting the service.

The quarterly general court of the governors of the Middlesex Hospital was held in the board-room of the hospital on Thursday, August 30th, at twelve o'clock noon. Mr. Treasurer Ruthven Pym presided, and there were present: The Acting Chaplain, Mr. Treasurer Hoare, Major Ross, M.P., Mr. Burroughes, Mr. Attenborough, Dr. Cayley, Dr. Fowler, Dr. Pringle, Dr. Edis, Dr. Duncan, Mr. Clark, Mr. Bendelow, Mr. Parr, and Miss Thorold. Dr. Robert Liveing, who has resigne dthe position of physician for skin diseases after holding it for twenty years, was unanimously appointed consulting physician. Mr. Augustus Pigott Oldershaw, as trustee of the late Miss Priscilla Sarah Bradley, was appointed an honorary governor. The following list of legacies was read: Sir Robert Loder, Bart., £1,000; Lady Buchan, £1,000; and Miss A. H. Cavenish Bentinck (Cancer Fund), £2,500. An emendation of the laws to be observed by the obstetric physicians was proposed and carried.

A story of child murder comes from Birmingham which is almost incredible. Two children, a boy twelve years old and his sister ten, are left by a mother in charge of their infant cousin. The mother returns to find one of the baby's feet almost severed from the body, and the other dangerously wounded with a cutting instrument. The girl confesses that somebody in the street had told her she would be lucky for life if she cut off the child's feet. Fortunately the baby died within a short time of the perpetration of the deed. What kind of training and supervision can this wretched girl have had to be capable of so dreadful a deed to a helpless infant? Above all, what is now to be done with her? She is clearly of such mental and moral organization as to be below any reasonable standard of sanity. She is probably in no sense a lunatic, but belongs rather to the idiot type. She must be dealt with accordingly. Punishment no doubt she must have, and that of a severe kind; but years of instruction of the highest kind can alone raise her to the level of ordinary humanity.

THE following vacancies are announced this week, the amount of salary in each case being given after the name of the institution:—

Belgrave Hospital for Children. Resident House Surgeon and Assistant Secretary.—£30 per annum, with board and residence.
Cheltenham General Hospital. Resident Surgeon for the Branch Dispensary.—£180 per annum, with partly furnished house.
East London Hospital for Children, Shadwell, E. Resident Clinical Assistant.—Board and lodging.
Edinburgh City Poorhouse, Craiglockhart. Resident Medical Officer.—£80, with board.
General Hospital, Birmingham. Assistant House Surgeon.—Board, residence, etc.
General Infirmary, Leeds. Resident Medical Officer and Pathologist.—£100, with board, residence, etc.
Manchester Royal Infirmary, Dispensary, and Lunatic Asylum. Honorary Assistant Physician.
Newport and County Infirmary and Dispensary, Newport, Mon. House Surgeon.—£100, with board and residence.
Newtownmount Kennedy Fever Hospital. Physician.
Rotherham Hospital. Assistant House Surgeon.—Board and residence.
Royal Hospital of Bethlem. Assistant Medical Officer.—£300 per annum, with furnished residence.
The General Infirmary, Hull. House Surgeon.—100 guineas, with board and furnished apartments.
University of Aberdeen. Chair of Chemistry.
Winchcomb Union (No. 2 District). Medical Officer.—£65, with fees.

THE management of the London Homœopathic Hospital and Medical School, Great Ormond Street, have decided to open a new convalescent home at Eastbourne. Already a large amount has been subscribed, including the following donations: Mrs. Clifton Brown, £1,000; Sir J. Alexander, £500; Mr. G. Sturge, £500; and Lord Grimthorpe, 100 guineas.

THE *Philadelphia Ledger* gravely assures its readers: "London has so many doctors in excess of her physical requirements, that in the poorer parts some of them will see a patient, prescribe, and supply medicine at sixpence a visit." There is a certain amount of truth in this statement, but those who inquire into the matter will usually find that the doctors who charge sixpence a visit have been driven from more remunerative practice through some fault of their own, and the fact that they are forced to be content with such small fees has nothing whatever to do with the proportion of doctors to patients.

THE passing of the Truck Act threatened to act injuriously on the workmen's contributions to the Belfast Royal Hospital, as the authorities felt it right to inform the principals of the firms who had contributed that they could not accept the money without receiving the written consent of every workman who subscribed. Rather than go to the trouble of obtaining this, several firms ceased making collections for the hospital, but the difficulty has been got over, and the proposal to form a board of workmen governors, to work conjointly with the others, promises to add to the efficiency of the hospital, and to add to its resources.

THE trustees of the People's Palace are holding a six weeks' autumn fête, the main attraction of which consists of a fine exhibition of modern pictures, under the management of the directors of the New Gallery. The price of admission during the day is fixed at twopence, and in the evening (after five) at one penny. This results in the attendance in large numbers of the poorer inhabitants of the East-end, more than 121,000 having passed the turnstiles during the first two weeks of the fête. Much appreciation of the beauties of the pictures is shown, and the concerts, illuminations, etc., which are held are also extremely popular. On Sundays, when the library is open free, and two organ recitals of sacred music are given in the Queen's Hall, these buildings, in which the pictures are hung, are attended by an average number of some 5,000 persons. These large numbers strikingly illustrate the success of the People's Palace, and warrant confident hopes of the equal success of the similar efforts now being made on behalf of South London.

A VERY handsome and commodious entrance porch and hall has recently been erected at the Leicester Infirmary as a memorial associated with the name of the late George Pearce, M.D., F.R.C.S., one of the honorary surgeons of that infirmary, who died on October 1st, 1886. It is a great boon to the infimary, especially benefiting the patients who, on being taken there, are now entirely under shelter and free from exposure, whilst architecturally it is in harmony with the main building. It contains two large and beautifully-executed stained glass windows, by Mr. H. L. Gardiner, of Leicester, one representing the figure of Æsculapius, with the words underneath: "This porch was erected in 1887 by friends of the late George Pearce, M.D., F.R.C.S., an hon. surgeon to this infirmary, in grateful remembrance. Born, 1839. Died, 1886." And the other representing the figure of Hygeia, with the word underneath: "The Leicester Infirmary for the reception and relief of the sick and poor from any county or nation. Established 1771." The entire cost of this memorial porch was £495 18*s.* 6*d.*, which has been subscribed for entirely by the friends and patients of the late Dr. Pearce. Mr. S. Francis Stone, of Kirby Frith Hall, near Leicester was treasurer and hon. secretary.

A LECTURE was delivered on August 24th by Staff-Surgeon Dickinson, at St. James's Hall, on Droitwich as a health resort, and the treatment and cure of rheumatism in all its varieties, together with gout, swelling of the joints, scrofula, etc., by means of the brine baths. Droitwich would also appear to be a very important spa for Anglo-Indians suffering from tropical debility and malarial cachexia. The brine baths also acted as a tonic, and those who had undergone the wear and tear of London life rapidly recovered their health and strength. The brine baths acted as a powerful stimulant to the skin, and promoted its nutrition. By its action on the actaneous nerves it gave tone to the system. Mr. Dickinson narrated some very remarkable cases that had been completely cured by a visit to Droitwich. Mr. John Corbett, M.P., who owned most of Droitwich, had spared no expense in rendering it one of the most complete health resorts in England. Epidemic was unknown in Droitwich, the air being antiseptic from the chlorine that escapes in the process of evaporation in the manufacture of salt. The air was dry, the temperature never very cold, the water perfectly pure, the rainfall below the average, and the scenery and drives lovely, making Droitwich equally suitable both as a summer and winter health resort.

AT the monthly meeting of the Manchester Infirmary Board, held on the 27th inst., Mr. W. O'Hanlon reported upon the cases of small-pox which had been received into Monsall Hospital from St. Joseph's Industrial School. He said that altogether 74 cases were received. Three had died, 47 had been cured, and the other 24 cases were convalescent, and in the course of a very few days would be dismissed. Of the 74 cases seven were unvaccinated and 67 vaccinated. All the vaccinated cases recovered, but three of the unvaccinated cases died. Mr. O'Hanlon also said that during the last few days two patients suffering from typhus fever had been received at Monsall from a miserable dwelling in Manchester, and, as they stated that they slept ten in a bed, he thought it advisable to make some further inquiries as to the truth of the statement and as to the conditions under which the cases arose. It seemed that the statement was perfectly correct. Ten children and a man and his wife all slept in the same room. The ages of the children, who were of both sexes, ranged from one year to seventeen years. The room in which they slept was 12 ft. by 9 ft., so that each of them had about 81 cubic feet of air. In Monsall 2,000 cubic feet of air for each patient was considered necessary for the preservation of health. This case, Mr. O'Hanlon added, threw some light on the causes of the high death-rate in Manchester. Nothing further was said on the subject.

